# Targeting cell-derived markers to improve the detection of invisible biological traces for the purpose of genetic-based criminal identification

**DOI:** 10.1038/s41598-023-45366-y

**Published:** 2023-10-23

**Authors:** Mathilde Recipon, Rémy Agniel, Johanne Leroy-Dudal, Thibaud Fritz, Franck Carreiras, Francis Hermitte, Sylvain Hubac, Olivier Gallet, Sabrina Kellouche

**Affiliations:** 1 Equipe de Recherche sur les Relations Matrice Extracellulaire‐Cellules, ERRMECe, (EA1391), Groupe Matrice Extracellulaire et Physiopathologie (MECuP), Institut des Matériaux, I-MAT (FD4122), CY Cergy Paris Université, Neuville sur Oise, France; 2 Institut de Recherche Criminelle de la Gendarmerie Nationale, Cergy-Pontoise, France

**Keywords:** Carbohydrates, DNA, Proteins, Biological models, Cytological techniques, Gene expression analysis, Genetic techniques, Genomic analysis, Imaging, Immunological techniques, Microscopy, Sensors and probes, Sequencing, Software, Biochemistry, Biological techniques, Biotechnology, Sequencing, DNA sequencing, Cell adhesion, Extracellular matrix, Focal adhesion, Integrins, Cell biology, Cytoskeleton, Actin, Intermediate filaments, Eukaryote, Genetic markers, Interspersed repetitive sequences, Genetics, DNA sequencing, Chromatin

## Abstract

At a crime scene, investigators are faced with a multitude of traces. Among them, biological traces are of primary interest for the rapid genetic-based identification of individuals. “Touch DNA” consists of invisible biological traces left by the simple contact of a person’s skin with objects. To date, these traces remain undetectable with the current methods available in the field. This study proposes a proof-of-concept for the original detection of touch DNA by targeting cell-derived fragments in addition to DNA. More specifically, adhesive-structure proteins (laminin, keratin) as well as carbohydrate patterns (mannose, galactose) have been detected with keratinocyte cells derived from a skin and fingermark touch-DNA model over two months in outdoor conditions. Better still, this combinatory detection strategy is compatible with DNA profiling. This proof-of-concept work paves the way for the optimization of tools that can detect touch DNA, which remains a real challenge in helping investigators and the delivery of justice.

## Introduction

The development of fast and reliable forensic methods is required in order to identify potential criminals and victims involved and preserve security and safety. Within this context, DNA profiling could help investigators. It is currently used worldwide and is valuable to investigators and judicial officers. The collection of a biological sample from a crime scene and the recovery of its DNA content represent crucial steps in the DNA profiling process.

However, some biological traces are invisible to the naked eye and cannot be detected using the usual tools (like Polilight®, Luminol or Bluestar®)^1–3^. This is especially the case when working with a few cells left when touching an object or a person, which results in so-called “touch DNA” samples. Touch DNA is obtained from shed skin cells and other biological material (body fluids, epithelial cells, etc.) transferred from a donor to an object or to another person during physical contact^4–6^. This category of traces represents the majority of samples analyzed in forensic genetic laboratories (French and Swiss national databases) and is considered to be an important tool for investigators.

The cellular origin (in addition to different donor status^7^) of touch-DNA samples is still under debate. One of the sources is epithelial cells shed from skin called corneocytes. Corneocytes are anucleate keratinocyte cells in terminal differentiation and are present in the superficial layers of the skin^8–10^. Other origins of touch DNA can be some residual cell fragments from dead cells^11–13^, cell-free DNA^14,15^, or some nucleated cells from biological fluids (saliva, nasal secretions, sebum, tears, etc.)^16,17^.

To get an informative DNA profile, that is to analyze the variation of the DNA between individuals, a crucial first step is the optimal collection of biological traces. Nevertheless, an important question concerning touch DNA remains: how can a maximum of genetic material be collected without seeing any biological trace? Unlike blood traces, touch DNA is difficult to detect and, currently, no satisfactory detection method is available in the field. Hence, these samples are often blindly collected by operators using a hypothetic-deductive approach to determine what object the suspect might have touched. This could result in collecting from a less relevant area, miss a major trace, and so lose the genetic material.

Visual DNA detection techniques are not as common and are therefore a drawback of touch DNA. Reported examples include the use of DNA dyes such as Diamond™ Nucleic Acid Dye (Promega, USA)^18–20^. These publications show the effectiveness of Diamond™ in the case of “classic” amplification. According to some authors, in the presence of Diamond™ genetic profiles show patterns of PCR inhibition^21^, while others do not^22,23^.

Depending on the composition of the touch DNA, DNA could be entrapped within cell-derived structures/fragments. Thus, in order to improve the collection of touch DNA, we have chosen to develop a trace detection strategy with either molecular targets (like DNA) but also with cellular targets known to be widely expressed by skin epithelial cells: 2 extracellular proteins (laminin and fibronectin), 1 transmembrane protein (integrin α5β1), 2 intracellular proteins (actin and keratin 10), and 5 carbohydrate patterns present in human cell glycoproteins (mannose, galactose, N-acetylglucosamin, and fucose)^24^.

This proof-of-concept study aimed to optimize the collection of touch DNA and thus improve detection by developing:

(i) Trace-DNA models: a first model with keratinocyte cell lines isolated from the skin in order to make the adjustments required and to determine the various thresholds of sensitivity (calibrated cell numbers, screening of molecular and cellular targets, DNA profile, etc.) and a second model based on contact of skin (fingers = fingermarks) with a glass slide;
(ii) A new strategy changing approaches to touch-DNA detection by targeting cell-derived fragments rather than DNA alone, which is compatible with genetic analysis;
(iii) The study of the persistence in time of biological traces and their detection as a function of environmental conditions.

## Results

An offence can take place not only indoors but also outdoors and the traces on the crime scene may not be detected immediately but several hours/days/months later. In order to be close as possible to the reality of a crime scene, touch DNA models (i.e. mock samples of keratinocyte cells and fingermark skin contact) were exposed to environmental climate conditions (temperature, hygrometric) and to the evolution of the traces over time.

### Detection and persistence of DNA over time

**Figure1:**
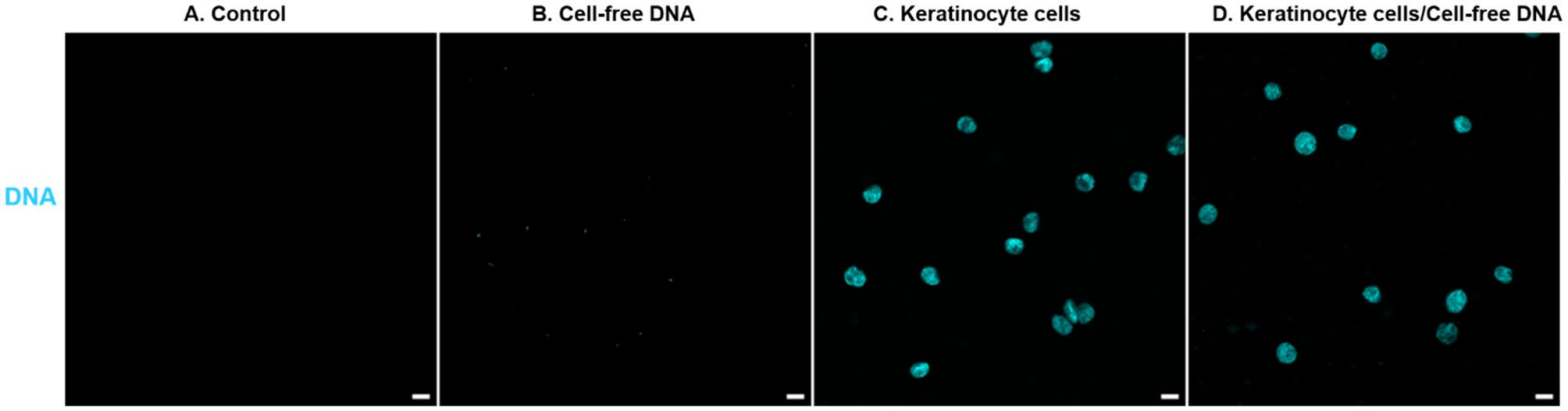
Detection of cell-free DNA with Hoechst 33342. Cell-free DNA (11.5 ng) and keratinocyte cells (5000/cm^2^) were deposited on glass slides. Control condition: culture medium alone was deposited. Cell-free DNA and cells nuclei were stained with DNA probe (Blue): Hoechst 33342.The image acquisitions are representative data from three independent experiments. Scale bar 10 µm.

In addition to corneocyte cells, some authors also mentioned that cell-free DNA could be the source of touch DNA^14^. In this study, the observation of cell-free DNA compared to cell DNA (nuclei) was examined with Hoechst 33342 (Fig. 1).

**Figure 2:**
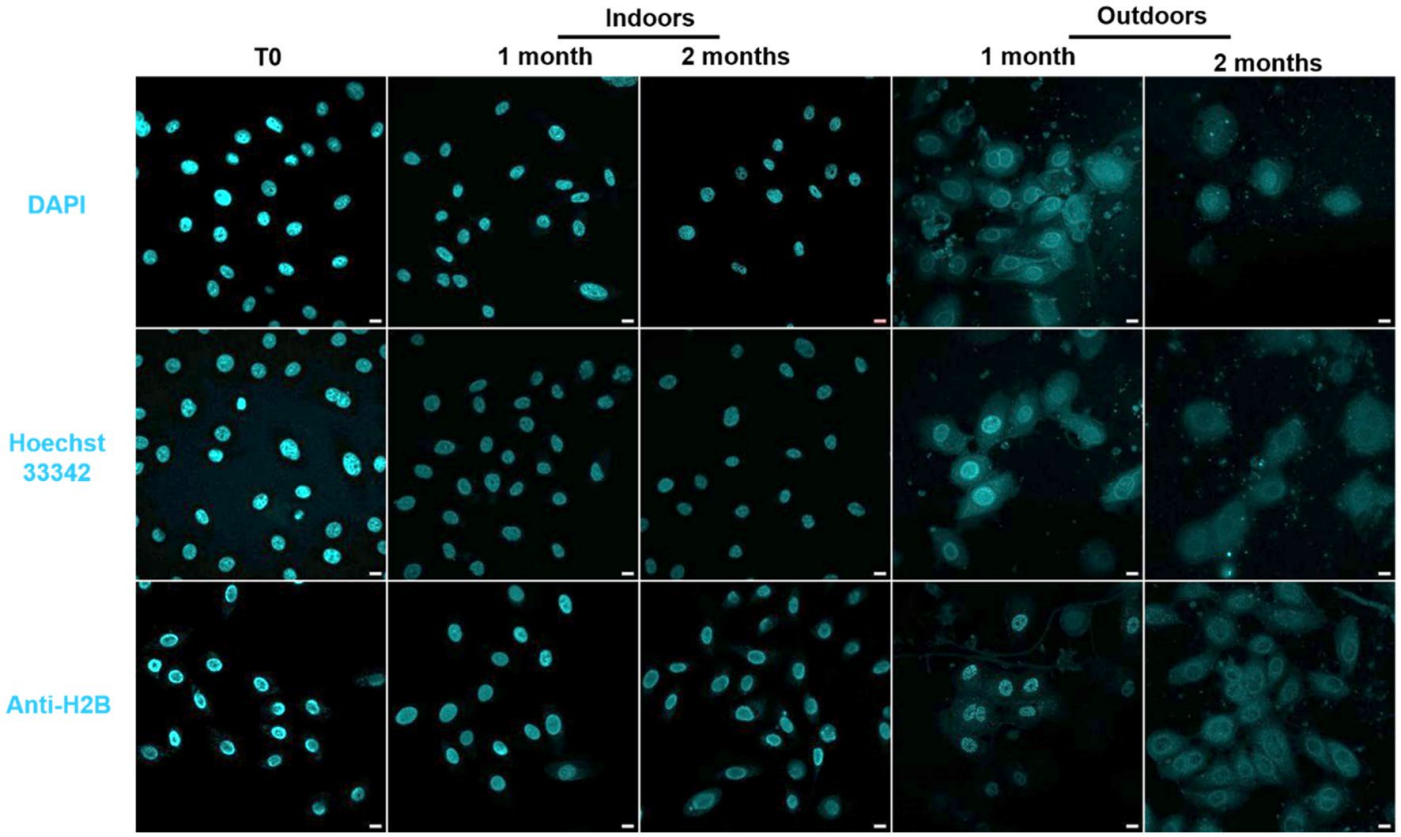
Detection and persistence of DNA labelling with DAPI, Hoechst 33342, and anti-H2B antibodies. Keratinocyte cells on glass slides were placed for up to 2 months indoors (in laboratory conditions, Table 1) and outdoors (in uncontrolled conditions according to the local climate). Cells nuclei were stained with DNA probes (Blue): DAPI, Hoechst 33342, or anti-H2B antibody. The image acquisitions are representative data from three independent experiments. Scale bar 10 µm.

Controls without cell-free DNA were negative. Cell-free DNA was not detectable with Hoechst 33342, only intracellular DNA was visualized. The targeting of keratinocyte cells’ DNA over time was tested with several specific DNA probes widely used in cell biology: DAPI, Hoechst 33342, and anti-H2B antibodies specific to histone H2B of eukaryote nuclei were used (Fig. 2).

Indoors, keratinocyte DNA was detectable during the two months of the experiment, whatever the nuclear probes tested. In outdoor conditions too, the genetic material of cells remained detectable with the three nuclear probes after 1 month. Even if staining could become more diffuse after 2 months, surprising apparent conservation of nuclei areas was observed. Thus, DNA could be detected over time during the experiments and was equivalently detected by the three probes. Among probes, Hoechst (33342) was selected as the detection probe for the following experiments because it was easy to use and time-saving.

### Detection and persistence of cellular protein targets over time

In order to enhance specific cell signals for touch-DNA detection, the possibility of combining the detection of cell-derived fragments with DNA probes was investigated.

**Figure 3:**
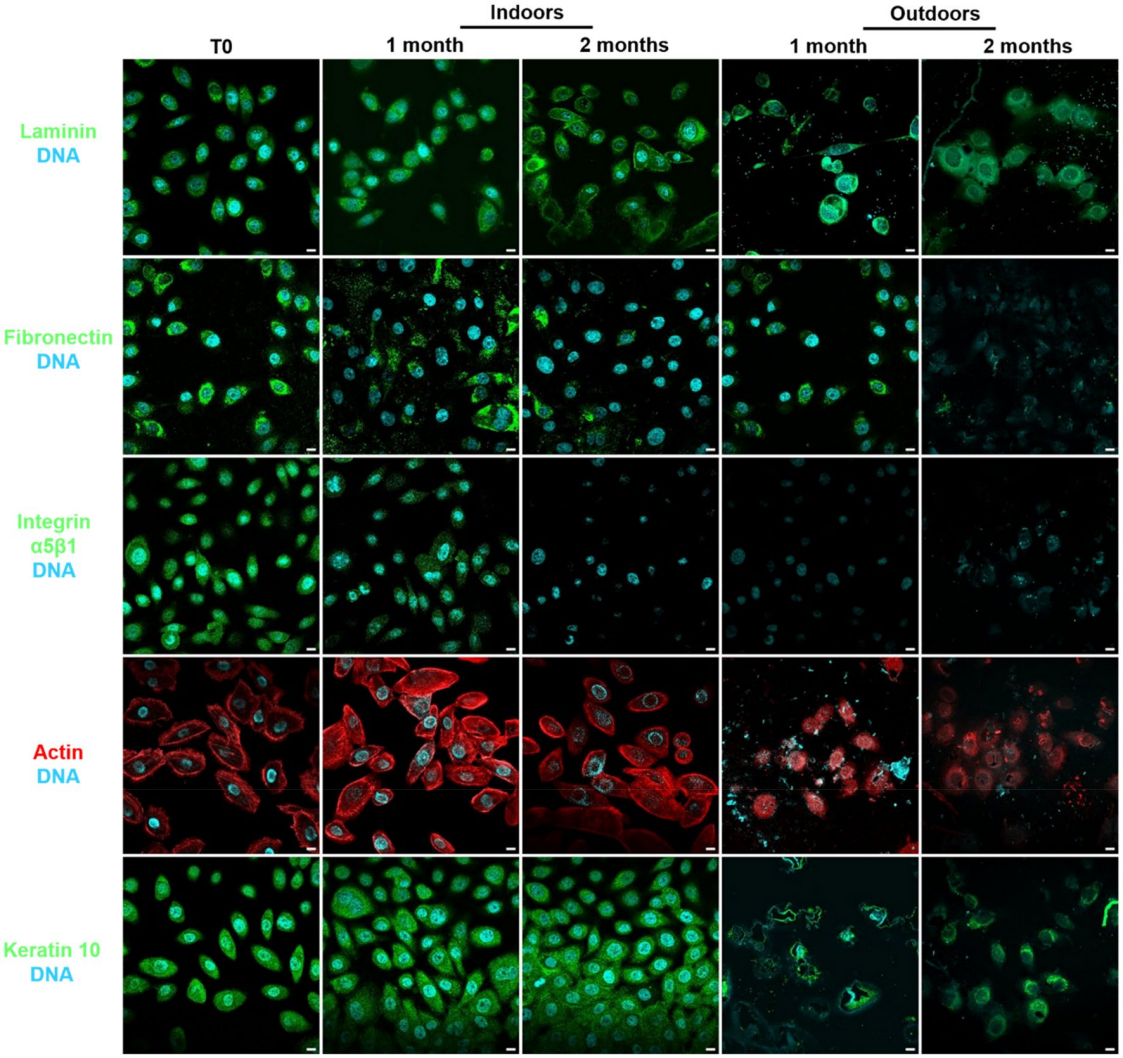
Detection and persistence of several cell protein targets on keratinocyte cells indoors and outdoors for up to 2 months. Keratinocyte cells on glass slides were placed for up to 2 months indoors and outdoors. Cells were labelled with antibodies specifically directed against laminin, fibronectin, α5β1 integrins, keratin 10 (Green), and phalloidin for actin (Red). In parallel, nuclear DNA was stained with Hoechst 33342 (Blue). The image acquisitions are representative data from three independent experiments. Scale bar: 10 µm.

The persistent presence of adhesive-structure proteins over time on keratinocyte cells placed indoors or outdoors (Table 1) for up to 2 months was observed using immunofluorescence (Fig. 3).

**Table 1:** Environmental conditions tested.

Samples	Indoors/outdoors	Temperature (average during two months)	Humidity (average during two months)
Cells	Indoors	19.0 °C ± 0.5 °C	50% ± 2%
	Outdoors	11.6 °C ± 3.3 °C	73% ± 5%
Fingermarks	Indoors	19.0 °C ± 0.5 °C	50% ± 3%
	Outdoors	6.5 °C ± 2.5 °C	90% ± 5%

Characteristic punctiform labelling of laminin was present around nuclei and persisted over 2 months in indoor and outdoor conditions. Fibronectin and α5β1 had a more diffuse labelling that faded gradually over time, especially in outdoor conditions. F-actin labelling was more diffuse but persisted for up to 2 months indoors and outdoors. Keratin 10 labelling was localized in the cytosol and near nuclei and persisted over time both indoors and outdoors.

Surprisingly, the staining of laminin, actin, and keratin 10 remained stable over time and resistant to environmental conditions. Moreover, nucleus DNA staining allowed the determination of the preservation or degradation of the apparent structure of nuclei when cells were placed indoors or outdoors, respectively.

### Detection and persistence of carbohydrate patterns over time

Another complementary target was the cell carbohydrates patterned by specific lectins. Lectins bind patterns harbored by cell glycoproteins. Four types of saccharides were targeted: mannose, galactose, N-acetylglucosamine, and fucose (Table 2).

**Table 2:** The principal carbohydrate patterns recognized by the 4 lectins tested.

Lectins	Species	Principal saccharides recognized
Con A	*Canavalia ensiformis*	Mannose (Man)
PNA	*Arachis hypogaea*	Galactose (Gal)
SNA	*Sambucus nigra*	N-acetylglucosamine (GlcNAc)
UEA	*Ulex europaeus agglutinin*	Fucose (Fuc)

**Figure 4:**
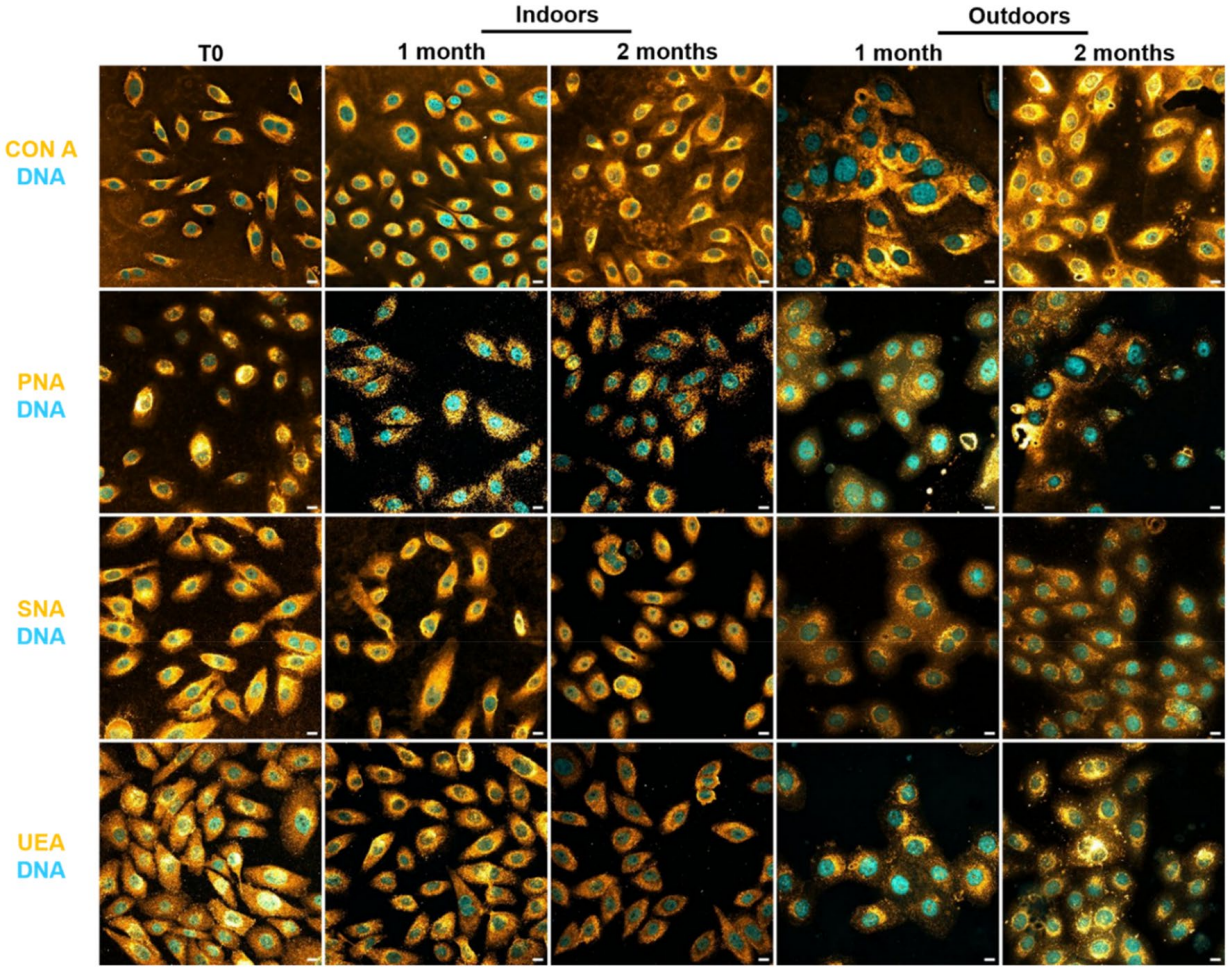
Detection and persistence of lectin targets on keratinocyte cells indoors and outdoors for up to 2 months. Keratinocyte cells on glass slides were placed for up to 2 months indoors and outdoors. Cells were incubated in the presence of Con A, PNA, SNA, and UEA lectins (Orange) that recognize specific carbohydrate patterns (Table 2). In parallel, nuclear DNA was stained with Hoechst 33342 (Blue). The image acquisitions are representative data from three independent experiments. Scale bar: 10 µm.

Cellular carbohydrates were targeted with biotinylated lectins, conjugated to fluorescent streptavidin (Fig. 4).

Con A, PNA, SNA, and UEA revealed punctiform and/or diffuse labels of carbohydrate patterns for up to 2 months indoors and outdoors. Nuclear DNA was detected indoors and outdoors, despite exposure to climatic variations. Furthermore, apparent nuclei structure was detectable after 2 months.

Carbohydrate patterns resistant to environmental conditions were efficiently revealed by the lectin approach using Con A, PNA, SNA, and UEA.

Thus, it seems interesting to deploy a combinatorial strategy allowing it to target laminin, keratin 10, and carbohydrate patterns with DNA for the purposes of optimizing touch-DNA detection (Table 3).

**Table 3:** Summary of the persistence of keratinocyte cell targets after 2 months indoors and outdoors.

Molecular type	Targets	Probes	Indoors	Outdoors
Nucleic acid	**DNA**	**Dapi**	** + **	** + **
	**DNA**	**Hoechst 33342**	** + **	** + **
Proteins	**Histone**	**Mab anti-H2B**	** + **	** + **
	**Laminin (Lam)**	**Pab anti-Lam**	** + **	** + **
	Fibronectin (Fn)	Pab anti-Fn	+	−
				*Disappears after 5 weeks*
	Integrin α5β1	Mab anti-5β1	−	−
			*Disappears after 5 weeks*	*Disappears after 4 weeks*
	Actin	Phalloidin	+	+
	**Keratin 10 (K10)**	**Pab anti-Ker**	** + **	** + **
Carbohydrate patterns	**Man**	**Con A**	** + **	** + **
	**Gal**	**PNA**	** + **	** + **
	**GlcNAc**	**SNA**	** + **	** + **
	**Fuc**	**UEA**	** + **	** + **

Table represents the persistence (+) or disappearance (−) of targets after 2 months indoors but also outdoors. *Pab* = polyclonal antibody, *Mab* = Monoclonal antibody. In bold, targets that were selected for further study because they persisted over time.

### Genetic profile edition after cellular target detection

**Figure 5:**
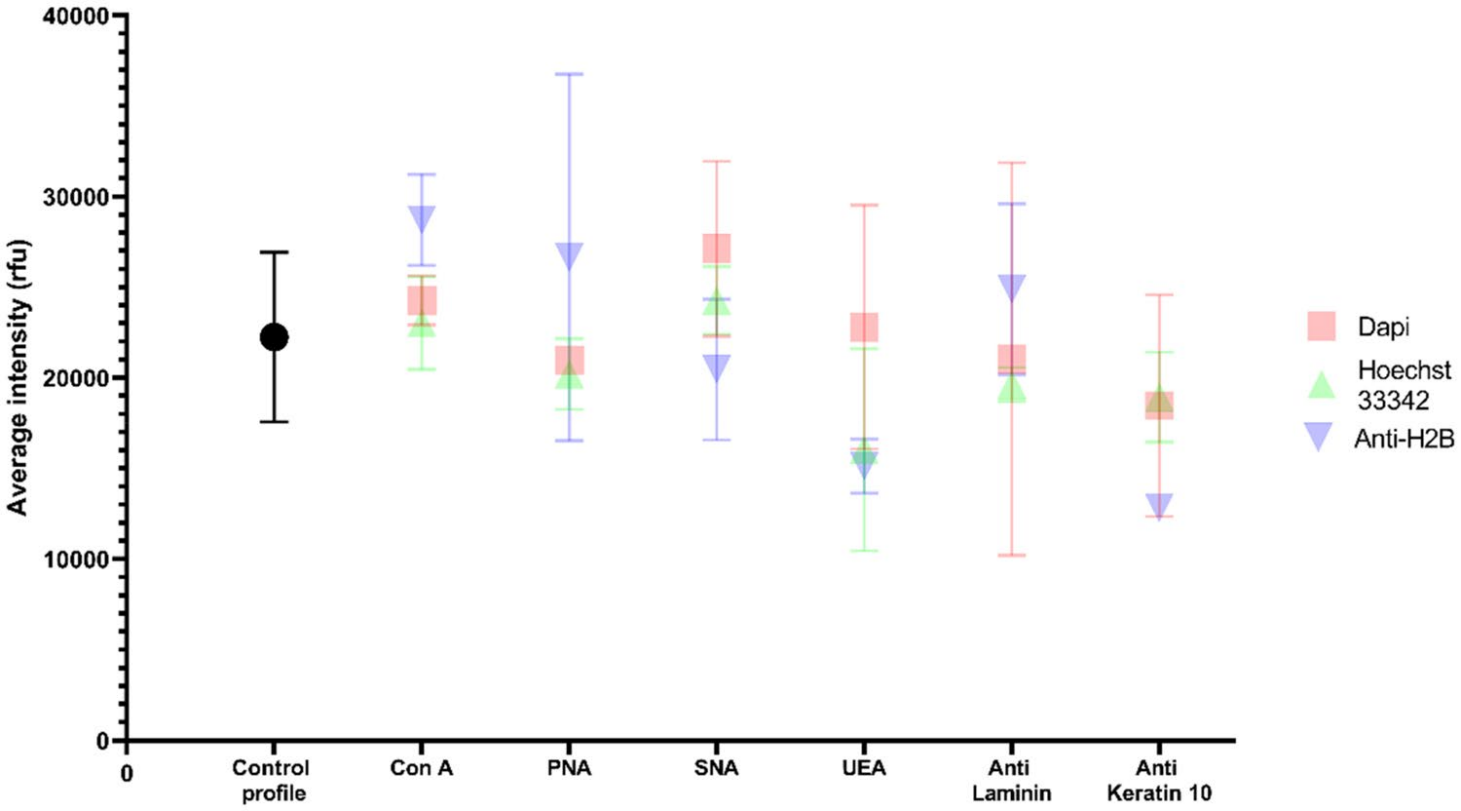
Average intensity of genetic profiles of keratinocyte cells. The sensitivity threshold was set at 50 rfu (internal laboratory conditions (IRCGN), determined and validated in quality assurance). The mean intensity of each profile as a function of probes was calculated for each experiment. Standard deviations were represented. The control profile was keratinocyte cells that were swabbed directly without any labelling. Data are representative of three independent experiments. P-values were calculated by one-way ANOVA test with no significant (ns) differences estimated by comparison control vs probes and probes vs probes.

Transposing this combined detection strategy into the field requires strict specifications, in particular no interference with the subsequent genetic analysis. Keratinocyte cells (Table 4, Fig. 5) were labelled with the different probes (antibodies, lectins, and DNA probes). Seven probes were chosen based on their ability to detect persistent molecular targets over time (Table 3). To target DNA, Dapi, Hoechst 33342, and anti-Histone H2B were selected. The probes that targeted the most persistent proteins over time are anti-laminin and anti-keratin 10. Phalloidin was not chosen because of its toxicity. The 4 lectins Con A, PNA, SNA, and UEA were also selected for labelling. The cells were then collected with a swab for genetic profile analysis (Table 4).

**Table 4:** Description of genetic profile of keratinocyte cells after cellular target detection.

		Keratincoyte cells				
		Usability of profile	Degradation index	Number of missing loci	Amelogenin	Q/S
Control		2/2	1.2 1	0/22 0/22	X–Y	Q/S
Dapi	Con A	2/2	0.8 1	0/22 0/22	X–Y	Q/S
	PNA	2/2	1.2 1.5	0/22 0/22	X–Y	Q/S
	SNA	2/2	1.2 1.2	0/22 0/22	X–Y	Q/S
	UEA	2/2	1 1	0/22 0/22	X–Y	Q/S
	Lam	2/2	0.8 1.3	0/22 0/22	X–Y	Q/S
	K10	2/2	1.1 0.8	0/22 0/22	X–Y	Q/S
Hoechst	Con A	2/2	1.2 1	0/22 0/22	X–Y	Q/S
	PNA	2/2	1.1 1.3	0/22 0/22	X–Y	Q/S
	SNA	2/2	0.9 1	0/22 0/22	X–Y	Q/S
	UEA	2/2	1.2 2	0/22 0/22	X–Y	Q/S
	Lam	2/2	1.1 0.9	0/22 0/22	X–Y	Q/S
	K10	2/2	1 0.9	0/22 0/22	X–Y	Q/S
Anti-H2B	Con A	2/2	1 1.1	0/22 0/22	X–Y	Q/S
	PNA	2/2	0.9 1	0/22 0/22	X–Y	Q/S
	SNA	2/2	1.1 0.9	0/22 0/22	X–Y	Q/S
	UEA	2/2	0.8 1.3	0/22 0/22	X–Y	Q/S
	Lam	2/2	1.2 1	0/22 0/22	X–Y	Q/S
	K10	2/2	1.5 1	0/22 0/22	X–Y	Q/S

The height of the peaks was measured in rfu (relative fluorescence unit): it was proportional to the quantity of PCR products detected. A profile was usable if the number of complete Short Tandem Repeat (STR) markers was greater than 5, according to the requirements of the French database *Fichier National Automatisé Empreintes Génétiques.* A profile was complete if all STR markers (22 STR) were amplified and present. Degradation index indicated the quality of the DNA (value close to 0: complete degradation; value close to 1: no degradation). Human gender determination was performed by analyzing the amelogenin gene which is common to both X and Y chromosomes. If the two quality sensors (Q and S) were visible on the electrophoregram, it meant that the PCR had been successful and there was no inhibition. 2/2 indicates the number of usable profiles per replicate. Results are representative of at least two independent experiments.

All profiles were usable, complete (22 STR), and expressed the internal quality controls (Q and S) which showed the absence of PCR inhibitors. DNA profiles had a degradation index close to 1, indicating that the DNA was intact and not degraded. No significant difference of fluorescence intensity was measured between the different probes used and control (Fig. 5).

In conclusion, cellular and molecular probes used for the detection of keratinocyte cells did not interfere with the genetic analysis, which favors their potential use in the field.

### Detection and persistence of fingermark samples over time

**Figure 6:**
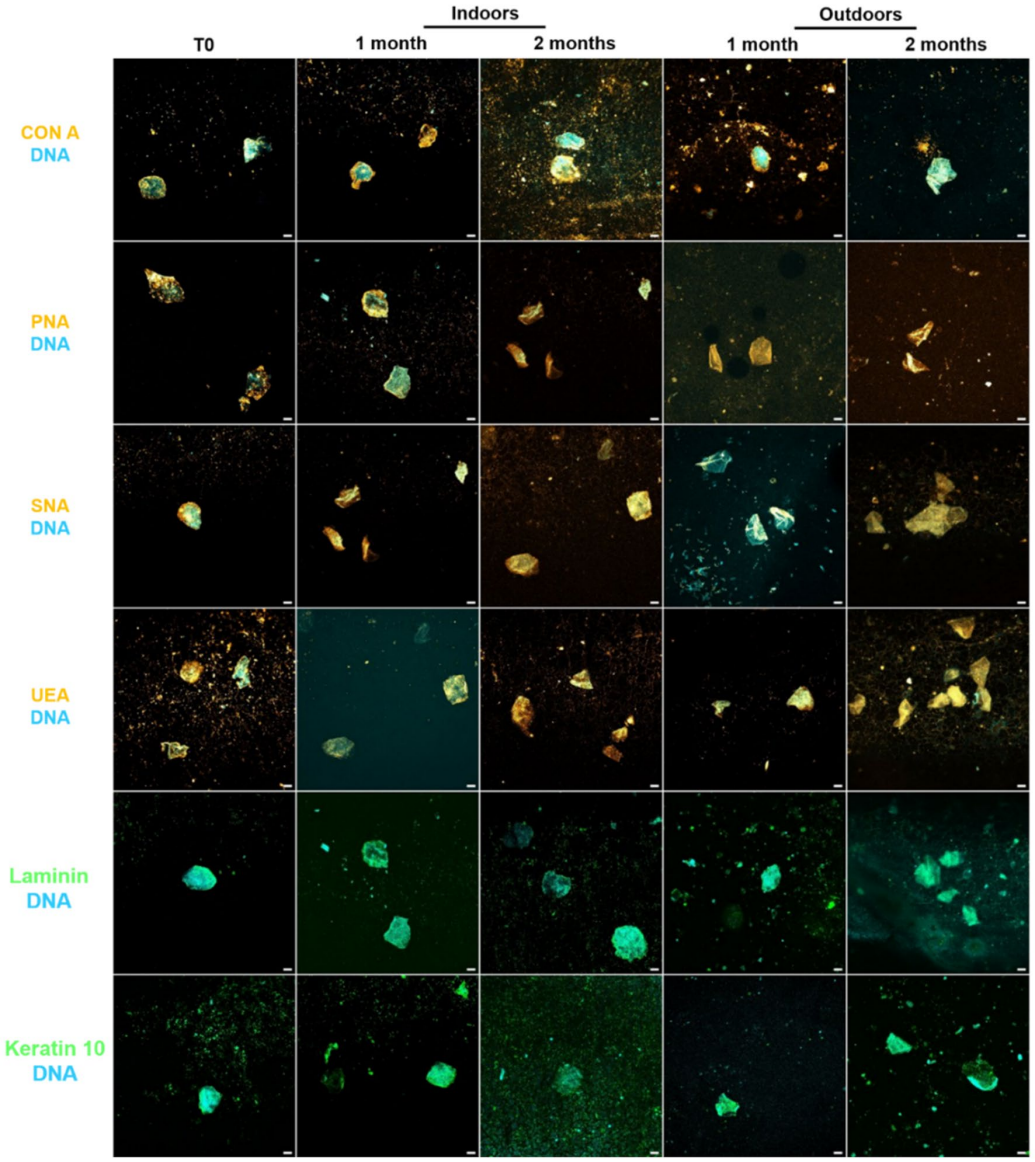
Detection and persistence of cell proteins and lectin targets on fingermarks indoors and outdoors for up to 2 months. Fingermarks were deposited on glass slides and incubated in the presence of Con A, PNA, SNA, and UEA lectins (Orange) that recognize specific carbohydrate patterns (Table 2) and antibodies specifically directed against laminin and keratin 10 (Green). In parallel, DNA from nuclei was stained with Hoechst 33342 (Blue). The glass slide was placed indoors or outdoors for 2 months. The image acquisitions are representative data from three independent experiments. Scale bar: 10 µm.

The seven targets (DNA probes, antibodies, and lectins) were then used to detect fingermark samples. For this purpose, fingermarks were made by depositing a 10-s contact on a glass slide^5^ and then placing it indoors and outdoors for up to 2 months (Fig. 6).

After 2 months indoors and outdoors, cells released from fingermarks could be revealed by both Con A and PNA lectins, as well as by anti-keratin 10 which revealed labelling at cell edges. SNA, UEA lectins, and anti-laminin antibodies could also detect cells after 2 months outdoors and indoors, but the labelling was more diffuse. DNA staining with Hoechst was diffuse in cells but could be seen even after 2 months.

Whatever the environmental conditions, fingermark samples could be specifically detected several weeks after deposition thanks to the use of the molecular targets selected.

In order to analyze the compatibility of the different probes used on fingermarks for the subsequent genetic analysis, skin cells were labelled with the seven probes selected (antibodies, lectins, and DNA probes) and then collected using a swab (Table 5).

**Table 5:** Description of genetic profile of fingermarks after cellular target detection.

		Fingermark samples				
		Usability of profile	Degradation index	Number of missing loci	Amelogenin	Q/S
Control		3/3	1.1 0.8 0.7	5/22 4/22 4/22	X-X	Q/S
Dapi	Con A	2/3	0.8 0.9 1.8	5/22 5/22 19/22	X-X	Q/S
	PNA	3/3	1.2 0.8 0.8	17/22 17/22 10/22	X-X	Q/S
	SNA	3/3	0.8 0.8 0.9	13/22 17/22 16/22	X-X	Q/S
	UEA	3/3	1.5 1 0.8	14/22 16/22 10/22	X-X	Q/S
	Lam	3/3	0.7 0.7 0.8	14/22 12/22 16/22	X-X	Q/S
	K10	2/3	0.9 0.8 0.7	15/22 14/22 20/22	X-X	Q/S
Hoechst	Con A	1/3	0.8 0.7 0.9	20/22 1/22 18/22	X-X	Q/S
	PNA	3/3	0.7 0.9 0.7	16/22 2/22 2/22	X-X	Q/S
	SNA	2/3	0.7 0.7 0.7	21/22 9/22 3/22	X-X	Q/S
	UEA	2/3	0.8 0.7 0.7	14/22 5/22 19/22	X-X	Q/S
	Lam	3/3	0.7 0.7 0.7	17/22 11/22 10/22	X-X	Q/S
	K10	1/3	0.9 0.7 0.7	18/22 9/22 22/22	X-X	Q/S
Anti-H2B	Con A	3/3	0.8 0.6 1	16/22 15/22 12/22	X-X	Q/S
	PNA	2/3	0.7 0.8 1	1/22 18/22 11/22	X-X	Q/S
	SNA	3/3	0.7 0.8 1.2	16/22 9/22 16/22	X-X	Q/S
	UEA	1/3	0.7 0.9 0.7	18/22 19/22 3/22	X-X	Q/S
	Lam	3/3	0.7 0.7 1	2/22 8/2 17/22	X-X	Q/S
	K10	0/3	0.7 0.7 0.8	20/22 18/22 18/22	X-X	Q/S

The height of the peaks was measured in rfu (relative fluorescence unit): it was proportional to the quantity of PCR products detected. A profile was usable if the number of complete Short Tandem Repeat (STR) markers was greater than 5, according to the requirements of the French database *Fichier National Automatisé Empreintes Génétiques.* A profile was complete if all STR markers (22 STR) were amplified and present. Degradation index indicated the quality of the DNA (value close to 0: complete degradation; value close to 1: no degradation). Human gender determination was performed by analyzing the amelogenin gene which is common to both X and Y chromosomes. If the two quality sensors (Q and S) were visible on the electrophoregram, it means that the PCR was successful and there was no inhibition. 3/3 indicates the number of usable profiles per replicate. Results are representative of at least three independent experiments.

Profiles showed more variability compared to those of keratinocyte cells, which is normal because the amount of DNA is lower in fingermark samples (Table 5). No complete profile could be obtained but 42 profiles out of 58 could be used. All profiles expressed the internal controls (Q and S) which indicated the absence of PCR inhibitors. The degradation index of all profiles was close to 1, indicating that the DNA was intact and not degraded.

**Figure 7:**
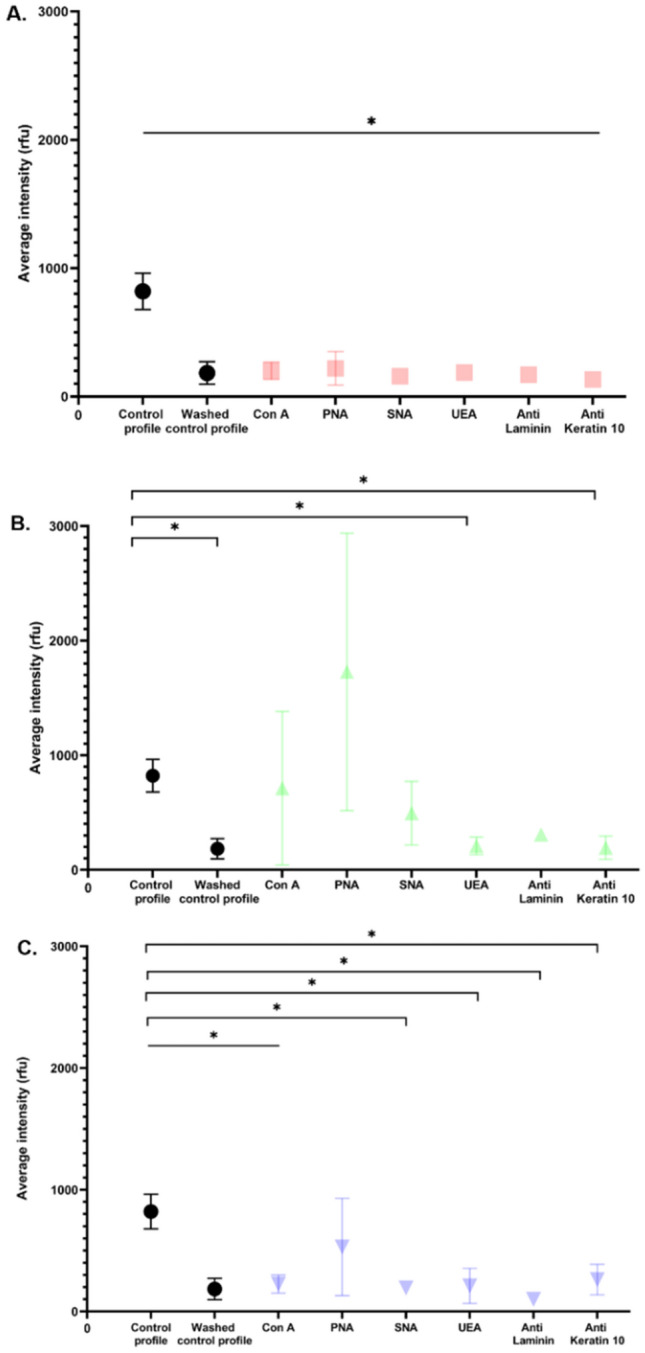
Average intensity of genetic profiles of fingermark samples. (**A**) Average intensity of genetic profiles of fingermark samples with DAPI. (**B**) Average intensity of genetic profiles of fingermark samples with Hoechst 33342. (**C**) Average intensity of genetic profiles of fingermark samples with anti-H2B. The sensitivity threshold was set at 50 rfu (internal laboratory conditions (IRCGN), determined and validated in quality assurance). The mean intensity of each profile as a function of probes was calculated for each experiment. Standard deviations were represented. Two controls were used for these experiments. The control profile was just a fingermark sample and collected directly by a swab. The washed control was a fingermark sample which was washed with PBS but not labeled, and then collected by a swab. P-values were calculated by one-way ANOVA test by comparison control vs probes and probes vs probes.

For Dapi and anti-H2B (Fig. 7A,C), it was observed that there was a significant difference between the control profile and the probes but not between the washed control and the probes. For Hoechst 33342 (Fig. 7B), only two significant differences were shown between the profile control and the UEA and anti-keratin 10 probes. For the other probes used with Hoechst 33342 (CA, PNA SNA and anti-laminin) no significant differences were noted with the control profile, nor with the washed control profile. There were no significant differences of fluorescence intensity between the different probes regardless of the DNA marker used (Fig. 7). These results suggest that the probes did not interfere with the genetic analysis.

### Detection of trace DNA on a manipulated mobile phone: proof-of-concept

In order to transpose the detection tools to the reality of a crime scene, DNA probes, antibodies, and lectins were used to detect touch DNA in situ. For this purpose, a mobile phone was manipulated for 10 s. A mixture of antibodies and lectins was then applied to an area. The revelation of these traces was done with the handheld device Crime-lite® 2 usually used by investigators on crime scenes.

**Figure 8:**
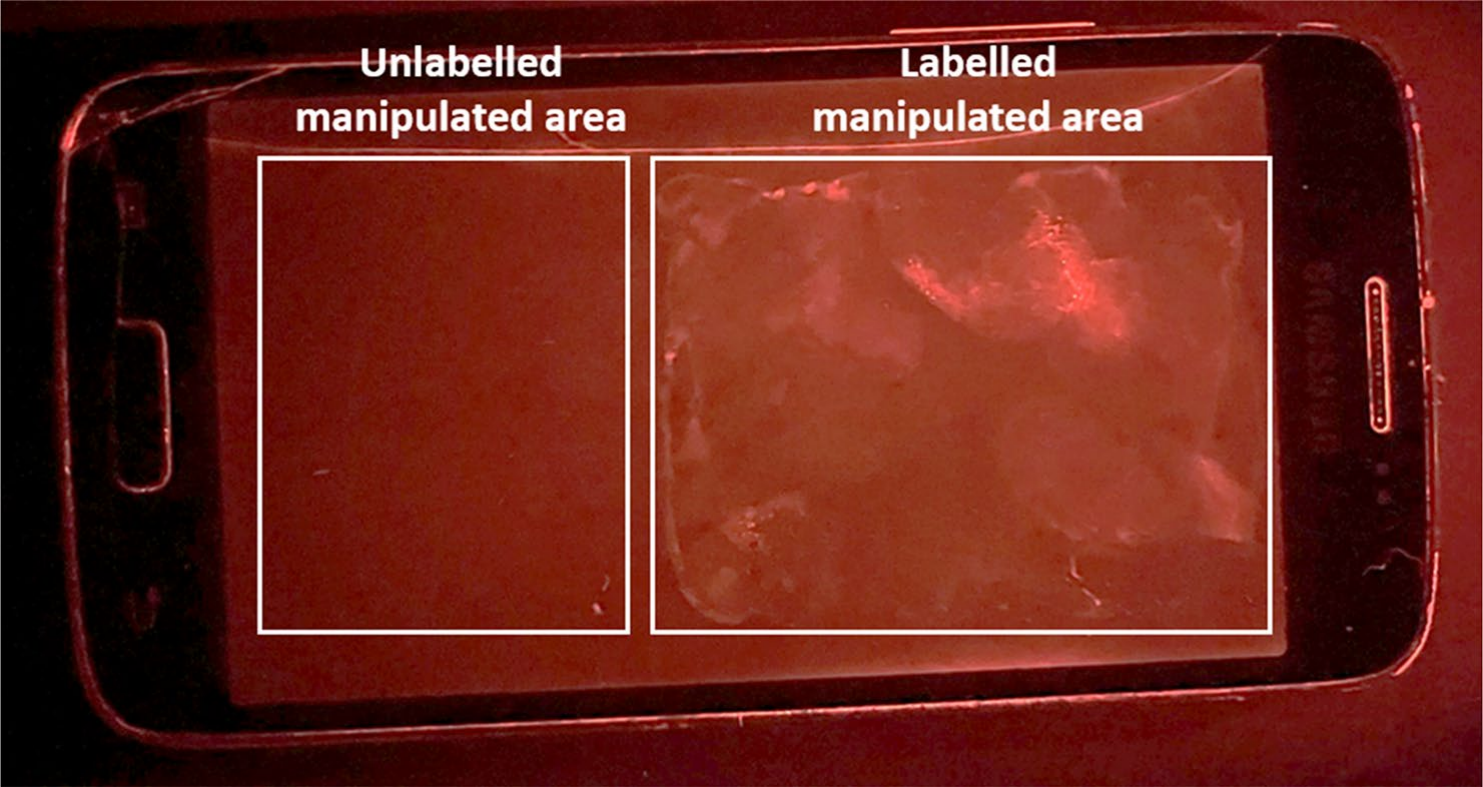
Detection of human cellular traces on a manipulated mobile phone with Crime-lite® 2. A volunteer manipulated a phone during 10 s. A mixture with DNA probe (Hoechst 33342), antibodies directed against laminin, against keratin 10, and the 4 lectins (ConA, SNA, PNA, and UEA) was applied (Right: labelled manipulated area; Left: unlabeled manipulated area). Fluorescence could be observed with Crime-lite®2 (450–510 nm and 490–560 nm). A picture was taken with camera filters.

A mixture with the most relevant antibodies (those directed against laminin and keratin 10) and the four lectins revealed molecular and cellular targets associated to human cellular traces on a mobile phone (Fig. 8). It was also noted that no fluorescence had been observed in the unlabeled manipulated area, which confirmed the specificity of the probes used.

Thus, these different relevant probes tested in this study could be used on crime-scene objects, and they revealed useful and important cellular traces allowing accessible DNA for future DNA profiling. The proposed concept will help investigators to visualize precise collecting zones in the bid to enhance swab recovering.

## Discussion

Our study, which acts as proof-of-concept, provides a novel approach to detecting DNA traces in forensic applications thanks to the unprecedented use of antibodies and lectins (in addition to DNA probes) targeting cell-derived structures. This approach is compatible with DNA profiling.

Many studies have been conducted to try to detect touch DNA but only with DNA probes such as Diamond™ Nucleic Acid Dye (Promega, USA)^19,20,25,26^. In the present study, in addition to DNA, a new combinatory approach was used, focusing also on cell structures and fragmented cell targets in order to maximize the chances of detecting touch DNA with lower toxicity in the field. In order to detect DNA, probes were chosen. The fluorescence emission of DAPI is very strong when it is bound to DNA, but it is toxic for users. On the other hand, Hoechst 33342 and the anti-histone H2B are less toxic. Moreover, H2B antibodies only recognize eukaryotic DNA.

Thus, the choice of original immuno- and lectino-fluorescence approaches combined with DNA probes makes it possible to target a large number of molecules (proteins, carbohydrates patterns, DNA) to increase the chances of detecting a maximum of touch DNA. These detection tools are also transposable to the detection of fingermark touch DNA (skin contacts) and manipulated objects in situ. The procedure could be rendered more suitable for forensic practice by directly spraying the relevant probes used onto crime-scene objects, leading to the detection of touch DNA for swabbing and future DNA profiling.

Studies of the stability and durability of cell targets over time are a major advance for the forensic world. Cellular targets or carbohydrate patterns were highly detectable for up to 2 months in an environment that was unfavorable to their preservation. It is also important to note that cells, placed outdoors for up to 2 months, were in contact with all kinds of contamination (microorganisms, pollen grains, etc.) but remained specifically detectable. Some cell structures were better preserved when cells were placed inside than when they were exposed outside. This can be explained by the environmental conditions (Table 1). Indeed, UV radiation will excite photosensitive endogenous compounds, inducing the production of reactive oxygen species (ROS).These will then damage cellular components such as lipids, proteins, or even DNA^27^. Humidity and heat will enhance microorganism proliferation causing DNA damage. Some studies show a direct correlation between the growth of bacteria and the resulting loss of DNA^28^. These reasons can explain the loss of some molecules of interest.

When a fingermark is deposited, cells appear to have no well-defined nuclei, but DNA probes still detect nucleic acids. Several hypotheses can be made: the degradation of organelles and the nucleus can release fragments or free nucleic acid residues in the corneocytes^9^, or possible DNA fragments bound to the corneocyte membrane recruited from nucleic acids which originate from sweat^14^ or extracellular DNA attached to the corneocyte membrane^15^.

The development of representative models of touch DNA is still lacking today. Laboratory models can provide the scientific community with a valuable tool to better understand the characterization of touch DNA. These models could pave the way for the optimization of their detection, sampling, and analysis.

The possible composition of touch DNA found in the literature has allowed the development of touch DNA models. Corneocytes from the skin surface are found particularly in touch DNA traces^9,16^. Corneocytes are epithelial cells derived from keratinocyte cells that have undergone terminal differentiation, resulting in a flattened, anucleate cell morphology (but still containing free DNA), without organelles and rich in keratin proteins (as with keratinocyte cells). However, corneocyte cells are not yet provided by conventional companies that produce cell lines. In our study, keratinocyte cells appeared to be the most appropriate epithelial cell model and were compared to fingermark touch DNA. One of the next challenges was to be able to adapt conventional cell culture techniques to unusual conditions, that is to maintain the requirements of cell culture while placing the cells in a more drastic environment (outside of conventional culture conditions in a sterile atmosphere, without a medium, in ambient air, and at varying temperatures). Cell adhesion and persistence were observed for several weeks (even if the cells were dead).

Today, although touch DNA samples represent approximately 80% of samples taken in criminal cases, only 30% will yield a usable DNA profile that complies with the requirements of the French database. It is therefore a major challenge for the forensic world to solve this problem in such a way that saves time and money.

This study could overcome these obstacles by proposing a less costly approach and new combined strategies for the detection of touch DNA in the forensic practice. The lack of interference from the detection tools used prior to the genetic analysis is a major asset in their deployment in the field.

## Conclusion

This study aimed to detect touch DNA even after a long period. For this purpose, an innovative combinatory detection strategy which consists of characterizing specific molecular and cellular targets of touch DNA was developed. The stability of the cellular and molecular targets (DNA, protein, and carbohydrate patterns) allows the detection of skin epithelial cells (keratinocytes) but also of fingermark touch DNA even after 2 months, not only indoors but also outdoors. The detection approach didn’t interfere with the genetic analysis. From this perspective, the proof-of-concept of a detection strategy and methodology was provided in vitro* and *in situ for operational development on a crime scene.

## Materials and methods

### Biological samples

#### Keratinocyte cells

A human keratinocyte cell line isolated from skin (CCD 1106 KERTr CRL-2309™) was purchased from ATCC® (USA) with a certificate of conformity. Cells were cultured in Keratinocyte-SFM medium with L-glutamine supplemented with 2.5 ml of Bovine Pituitary Extract BPE (Thermofisher, USA) and 35 ng/ml of Human Recombinant EGF (VWR, USA). Cells were cultured under humidified atmosphere at 37 °C and 5% CO_2._ To set up our detection strategy, cells were placed on glass slides at 5000/cm^2^. After 4 h of incubation, the medium was removed and adherent cells were left to dry for up to 2 months.

#### Cell-free-DNA sample preparation

To get cell-free DNA, a tube containing a pellet of 300,000 keratinocyte cells was added to 400µL of Crime Lysis Buffer, 50µL of Proteinase K, and 4.5 µL of 3 M Dithiothreitol (Crime Prep, Adem-Kit AutoMag solution, Ademtech, France). The tube was placed in a thermomixer and incubated at + 56 °C at 500 rpm for 40 min. After 2 min of centrifugation at 3500 rpm, DNA was extracted with a KingFisher DUO (Thermofisher, USA) according to the manufacturer’s protocol.

To quantify the different DNA extracts, a NanoDrop One (Thermofisher, USA) was used. A 2 µl drop of the extract was deposited and measured. A concentration in ng/µl was then calculated.

To establish our strategy detection^14^:

- 11.5 ng of cell-free DNA was deposited on glass slides.
- A mixture of keratinocyte cells and 11.5 ng of cell-free DNA were placed on glass slides.

Samples were stained 30 min at room temperature in the dark with Hoechst 33342 (H1399, Thermofisher, USA) diluted 1:3000 in Phosphate Buffered Saline-Bovine Serum Albumin (PBS-BSA) 0.5%. Slides were mounted with Prolong mounting medium (P36930, Invitrogen).

#### Skin contact samples: fingermark touch-DNA model

All samples were obtained with the informed consent of the volunteers and the approval of the Ethics Committee of the Pôle Judiciaire de la Gendarmerie Nationale (PJGN), in accordance with the Helsinki agreements (1975) and the French National Charter for Research Integrity. The PJGN Ethics Committee issued recommendations which were followed for this study.

Skin contact samples (named fingermarks) were placed on a glass slide that was previously cleaned with DNA-free water (Thermofisher, USA) and 70% ethanol. Fingermarks obtained from volunteers were collected using moderate pressure during 10 s^29^.

### Environmental conditions for keratinocyte cells and fingermark deposition

To mimic crime-scene conditions, both keratinocyte cells and fingermarks were placed indoors at room temperature (on the bench of the laboratory) and outdoors (on the windowsill) for up to 2 months in two different seasons (Table 1).

### Proteins and DNA fluorescence staining

After each time (T0, 1 and 2 months), keratinocyte cells were permeabilized with PBS-Triton X-100 0.1% for 4 min at 4 °C. Non-specific sites of keratinocyte cells and fingermark samples were blocked 30 min with phosphate buffered saline-Bovine serum albumin 0.5% at room temperature and incubated for 2 h with primary antibodies targeting extracellular or intracellular structures (all diluted 1:50 in PBS-BSA 0.5%): laminin (L9393, Sigma-Aldrich, USA), fibronectin (F3648, Sigma-Aldrich, USA), α5β1 (Mab 1969, Millipore, USA), and keratin 10 (SAB4501656, Sigma-Aldrich, USA). After washes with PBS, samples were incubated with the appropriate fluorescent secondary antibodies for 1 h in the dark: Alexa Fluor 488 conjugated anti-mouse antibody (A11029, Life Technologies, USA) and Alexa Fluor 488 conjugated anti-rabbit antibody (A11008, Life Technologies, USA) (all diluted 1:400 in PBS-BSA 0.5%). The F-actin was stained with TRITC-phalloidin (P1981, Sigma Aldrich). Thereafter, samples were stained 30 min at room temperature in the dark with DNA probes (all diluted in PBS-BSA 0.5%): DAPI (4',6-diamidino-2-phenylindole dihydrochloride, D9542, Sigma Aldrich) diluted 1:3000 and Hoechst 33342 (H1399, Thermofisher, USA) diluted 1:3000. For anti-histone H2B (SAB450223, Sigma-Aldrich, USA), it was incubated for 2 h (diluted 1/250 in PBS-BSA 0.5%). These samples were then incubated with the appropriate fluorescent secondary antibodies for 1 h in the dark (Alexa Fluor 488 conjugated anti-mouse antibody (A11029, Life Technologies, USA)).

Slides were mounted with Prolong mounting medium (P36930, Invitrogen). Controls without primary antibodies were negative.

### Carbohydrate patterns staining

Keratinocyte cells were recovered at each time point (T0, 1 and 2 months) and permeabilized with PBS-Triton 0.1% for 4 min at 4 °C. Keratinocyte cells and fingermark samples were saturated in PBS-BSA 1% for 30 min at room temperature. Samples were incubated for 20 min in a humidity chamber using a biotinylated lectin solution (at final concentration 10 µg/ml): Concanavalin A (ConA), Peanut agglutinin (PNA), Elderberry lectin (SNA), and Ulex europaeus agglutinin (UEA) (B-1005, B-1075, B-1305, and B-1065 respectively, Vector Laboratories, USA). After washing with PBS, samples were incubated with Alexa Fluor 555 Streptavidin (S21381, Thermofisher, USA), diluted 1:600, for 20 min in a dark chamber. DNA revelation of the nuclei was done using DNA probes: DAPI or Hoechst 33342 both diluted 1:3000 in PBS-BSA 0.5%. With anti-histone H2B (SAB450223, Sigma-Aldrich, USA), samples were incubated for 2 h (diluted 1/250 in PBS-BSA 0.5%) and then they were incubated with the appropriate fluorescent secondary antibodies for 1 h in the dark (Alexa Fluor 488 conjugated anti-mouse antibody (A11029, Life Technologies, USA)).

Slides were mounted with Prolong mounting medium. Controls without primary antibodies were negative.

### Microscopy analysis

Fluorescent labelling was observed with a confocal microscope (LSM710, Zeiss, Germany) equipped with a × 40 apochromatic oil immersion objective (NA: 1.2). The different analyses were conducted using the Zen 2010 BSP1 software (Zeiss, Germany). Acquisitions were performed under exactly the same conditions. The excitation wavelength for Dapi/Hoechst 33342 was set at 405 nm and collected between 421 and 517 nm. The excitation wavelength for Alexa Fluor 488 was set at 488 nm and collected between 494 and 552 nm. The excitation wavelength for Alexa Fluor 555 Streptavidin was set at 561 nm and collected between 565 and 680 nm. The pinhole aperture was set at one Airy Unit (AU). Images were processed with the Image J® software version 2.1.0 (National Institutes of Health, USA).

### Crime-lite® 2 analysis of handled object: Proof-of-concept

A mobile phone was manipulated by a volunteer during 10 s. Two types of area were set: one area manipulated but without labelling, and another area manipulated with labelling. Each area was saturated in PBS-BSA 1% for 30 min at room temperature. Samples were incubated in a humidity chamber with a mixture of biotinylated lectin solution (Con A, PNA, SNA, and UEA) and primary antibodies (laminin and keratin) for 1 h.

After washing with PBS, samples were incubated with Alexa Fluor 555 Streptavidin (S21381, Thermofisher, USA) and Alexa Fluor 488 conjugated anti-rabbit antibody (A11008, Life Technologies, USA) (all diluted 1:400 in PBS-BSA 0.5%) for 45 min in a dark room.

In order to visualize the different staining on the mobile phone, a handheld device named Crime-lite®2 (Foster + Freeman, UK), usually used on crime scenes, was applied in a black room. A Crime-lite®2 with a wavelength between 450 and 510 and another with a wavelength between 490 and 560 were used. Camera filters were used (OG550 and OG590 respectively) to take images.

### DNA profiling

#### Collection from cell and fingermark samples

Keratinocyte cells or fingermark samples were labelled with the different probes (antibodies, lectins, and DNA probes) and then collected from the glass slides using a swab (microFLOQ® Direct swabs, Copan, Italy). Each swab was moistened with 1 µl of deionized water and applied to the entire area where cellular material was deposited. After collection, swabs were left to dry for 15 min at room temperature before the direct DNA amplification step. With direct PCR, no extraction or quantification steps are required upstream of amplification.

#### DNA amplification

After collection, the head of microFLOQ® was broken directly into PCR strip tubes and amplified using Investigator 24plex QS (382415, Qiagen, USA). Investigator 24plex kits, which are for human identification, allowed the multiplex amplification of the CODIS (Combined DNA Index System) core loci, ESS (European Standard Set) markers, as well as SE33, DYS391, and Amelogenin markers. Kits included quality sensors (Q and S) to generate additional information about PCR success (such as whether amplification was successful, whether DNA was present in the sample, or whether inhibitors in the sample inhibited PCR). 15 µl of molecular-grade water and 10 µl of PCR mix were added directly to the tubes and DNA amplification was done immediately with an Applied Biosystems™ Veriti™ Thermal Cycler (Applied Biosystems™ 4375305 by Thermofisher, USA) during 28 cycles according to the manufacturer’s protocol.

#### Genotyping

Genotyping was performed using an ABI 3500xL genetic analyzer (Thermofisher, USA). Labelled PCR products (primers used during amplification are labelled during their synthesis and are then integrated into the DNA fragments to obtain labelled PCR products), along with a size control, were transferred into the capillary by electrokinetic injection at 1.2 kV for 24 s. After the injection, constant voltage was applied across the capillary and the PCR products migrated and were separated through capillary electrophoresis on the ABI 3500xL genetic analyzer, being detected by a laser depending on their molecular weight (base pair unit). Raw data generated at the end of electrophoresis were analyzed using GeneMapper ID-X v1.6 software to generate an electropherogram (EPG) that was a graphic representation of the DNA profile obtained.

#### Genetic profile analysis

On the EPG, each allele detected for each marker was represented by a peak with a height measured in rfu (relative fluorescence unit). The height was proportional to the quantity of PCR product detected and so the quantity of DNA. The detection threshold was set at 50 rfu in order to be able to detect everything that was amplifiable without it being background noise. Detection threshold was set at 50 rfu according to an internal validation of the IRCGN (from a source of DNA of known quantity and known STR profile, amplifications at 28 cycles were carried out by decreasing the initial quantity of DNA until obtaining a genetic profile with a true allele without artifacts). A reference genetic profile was established prior to the experiments. This preliminary study made it possible to define the fluorescence intensity thresholds of the alleles used to characterize a homozygous and heterozygous marker. A marker can be characterized as homozygous if a single allele is detected at a fluorescence intensity greater than 400 rfu, while the interpretation threshold for a heterozygous marker is set at greater than 150 rfu. Each genetic profile of the samples used was compared with the reference genetic profile.

The different genetic profiles were judged according to several criteria:

- Genetic profile usability: a genetic profile was declared usable and analyzable if the number of human STR markers was greater than 5. Otherwise it was declared unusable and not considered.
- Quality sensors Q/S: the presence of these controls showed the good efficiency of the PCR. In the absence of a quality control, PCR inhibition was detected.
- Degradation index (DI): this index was calculated by dividing the QS1 by the QS2. If the index was close to 1, profile did not show degradation. If the index was close to 0, profile presented degradation.

### Statistical analysis

The different results obtained are presented as the mean + / −  standard deviation of at least three independent experiments. Statistical analysis was made using the GraphPad Prism® Software (Dotmatics, USA). A one-way analysis of variance (ANOVA) test was used to assess differences. Statistical significances are designated by * indicating a two-tailed *p* < 0.05, **p < 0.01, and ***p < 0.001.

### Supplementary Information

Supplementary Table 1.

## Data Availability

The STR markers analyzed in this study are available in the GenBank® repository, [https://www.ncbi.nlm.nih.gov/genbank] as well as in the supplementary data, Table 1. The raw data from this study are available from the authors, but according to ethical approval by the PJGN (Pôle Judiciaire de la Gendarmerie Nationale, which issued recommendation n°10 of 12/03/2021 associated with a practical guide), restrictions apply to the availability of these data, which are not accessible to the public, as they involve sensitive data (genetic identification). They are stored in a secure database belonging to the French Gendarmerie Nationale. However, the sensitive data (related to the genetic profile of fingermarks) are available from Dr. Mathilde Recipon (mathilde.recipon@cyu.fr) or Dr. Sabrina Kellouche (sabrina.kellouche@cyu.fr) on reasonable request and with permission from the PJGN Ethics Committee.
