# Supplementary material for: Targeting cell-derived markers to improve the detection of invisible biological traces for the purpose of genetic-based criminal identification
Source: Sci Rep. 2023 Oct 23;13:18105. doi: 10.1038/s41598-023-45366-y (PMC10593828; doi:10.1038/s41598-023-45366-y)
Supplement: Supplementary file 1 — Supplementary Table 1. [file 41598_2023_45366_MOESM1_ESM.docx]

| Locus | Registration number GenBank® | Reference allele repeat motif |
| --- | --- | --- |
| Amelogenin X | M55418 | - |
| Amelogenin Y | M55419 | - |
| DYS391 | AC011302 | [TCTA]11 |
| D1S1656 | NC_000001.9 | [TAGA]16 [TGA][TAGA][TAGG]1[TG]5 |
| D2S441 | AL079112 | [TCTA]12 |
| D2S1338 | G08202 | [TGCC]6[TTCC]11 |
| D3S1358 | 11449919 | TCTA [TCTG]2 [TCTA]15 |
| D5S818 | G08446 | [AGAT]11 |
| D7S820 | G08616 | [GATA]12 |
| D8S1179 | G08710 | [TCTA]12 |
| D10S1248 | AL391869 | [GGAA]13 |
| D12S391 | G08921 | [AGAT]5 GAT [AGAT]7 [AGAC]6 AGAT |
| D13S317 | G09017 | [TATC]13 |
| D16S539 | G07925 | [GATA]11 |
| D18S51 | L18333 | [AGAA]13 |
| D19S433 | G08036 | AAGG [AAAG] AAGG TAGG [AAGG]11 |
| D21S11 | AP000433 | [TCTA]4 [TCTG]6 [TCTA]3 TA [TCTA]3 TCA [TCTA]2 TCCATA [TCTA]11 |
| D22S1045 | AL022314 | ATT]14 ACT [ATT]2 |
| CSF1PO | X14720 | [AGAT]12 |
| FGA (FIBRA) | M64982 | [TTTC]3 TTTTTTCT [CTTT]13 CTCC [TTCC]2 |
| SE33 (ACTBP2) | NG000840 | [AAAG]9 AA [AAAG]16 |
| TH01 (TC11) | D00269 | [TCAT]9 |
| TPOX | M68651 | [AATG]11 |
| vWA | M25858 | TCTA [TCTG]4 [TCTA]13 |

**Table 1 : Locus-specific information for Investigator 24plex kits**

**Supplementary Data**
